# Mandibular Arch Morphology in Normodivergent Patients With Impacted Maxillary Canines: A Case‐Control Study

**DOI:** 10.1155/ijod/8335110

**Published:** 2026-05-26

**Authors:** Mauro Lorusso, Michele Tepedino, Luigi Pio Marra, Elena D’Angelo, Fariba Esperouz, Lucio Lo Russo, Domenico Ciavarella

**Affiliations:** ^1^ Department of Clinical and Experimental Medicine, Dental School of Foggia, University of Foggia, Foggia, Italy, unifg.it; ^2^ Department of Biotechnological and Applied Clinical Sciences, Dental School of L’Aquila, University of L’Aquila, L’Aquila, Italy, univaq.it

**Keywords:** impacted canine, mandibular arch form, morphometric analysis

## Abstract

**Introduction:**

The morphologic characteristics associated with maxillary canine impaction have been extensively investigated at the maxillary level, whereas the contribution of mandibular arch morphology remains less explored. The purpose of this study was to evaluate mandibular arch form in patients with impacted maxillary canines.

**Methods:**

The present study included 96 normodivergent Class I subjects aged 10–12 years, divided into three groups: patients with buccally impacted maxillary canines (*n* = 32), patients with palatally impacted maxillary canines (*n* = 32), and a control group with physiologic canine eruption (*n* = 32). Mandibular arch morphology was assessed using an angular morphometric analysis performed on digitally acquired mandibular models. A reference polygon was defined using the cusp tips of the mandibular canines and the distobuccal cusps of the mandibular first molars, and the sums of its anterior and posterior angles were calculated. Cephalometric variables were used to control for sagittal and vertical skeletal relationships. Multivariate and univariate statistical analyses were performed to evaluate intergroup differences.

**Results:**

Multivariate analysis of variance (MANOVA) revealed a significant overall effect of group membership on mandibular morphologic variables (*p* < 0.001). ArGoMe values were 123.35 ± 7.67 in the palatal impaction group, 128.59 ± 8.83 in the buccal impaction group, and 127.47 ± 6.77 in the control group; anterior angle sums were 133.41 ± 6.97, 123.68 ± 11.17, and 135.07 ± 6.65, respectively; posterior angle sums were 217.47 ± 8.24, 226.07 ± 7.32, and 225.25 ± 6.95, respectively. Significant intergroup differences were found for ArGoMe (*p* = 0.013; partial *η* ^2^ = 0.090), anterior angle sum (*p* < 0.001; partial *η* ^2^ = 0.264), and posterior angle sum (*p* < 0.001; partial *η* ^2^ = 0.223), whereas no significant differences were observed for SNGoMe (*p* = 0.571; partial *η* ^2^ = 0.012). Post hoc analyses showed that the palatal impaction group had significantly lower ArGoMe values than the buccal impaction group (*p* = 0.014), and significantly lower posterior angle sums than both the buccal impaction and control groups (both *p*  < 0.001). The buccal impaction group showed significantly lower anterior angle sums than both the palatal impaction and control groups (both *p*  < 0.001).

**Conclusions:**

Mandibular arch morphology differs between patients with impacted maxillary canines and subjects with physiologic eruption. Within the selected normodivergent sample, these differences were observed without significant intergroup differences in vertical divergence. Distinct mandibular morphologic characteristics were associated with palatal and buccal canine impaction, suggesting that mandibular arch assessment may complement conventional diagnostic evaluation in patients with impacted maxillary canines.

## 1. Introduction

Dental impaction is defined as the failure of a tooth to erupt into its correct functional position within the dental arch despite the completion of root development and loss of eruptive potential [1]. Among impacted teeth, the permanent maxillary canine is one of the most commonly affected, with a reported prevalence of 1.0%–2.5% in the general population [2]. Approximately 8%–10% of affected individuals present with bilateral impaction [3]. Maxillary canine impaction occurs approximately twice as frequently in females as in males [4]. Palatal impaction is significantly more prevalent than buccal impaction, with a reported ratio of nearly 3:1 [5]. Although the overall incidence of impacted maxillary canines appears relatively consistent across ethnic groups, marked differences in localization have been reported. In Caucasian populations, palatal displacement accounts for ~85% of cases, whereas in Asian populations, buccal displacement is reported to be three to six times more frequent [6].

Several etiologic factors have been associated with maxillary canine impaction, including a prolonged eruption path, root dilaceration, inadequate arch space, dentoalveolar trauma, and cystic lesions [7]. However, the biologic mechanisms underlying canine impaction remain incompletely understood. Two principal theories have been proposed. The guidance theory suggests that agenesis, hypoplasia, or morphologic abnormalities of the maxillary lateral incisor may deprive the erupting canine of proper guidance, resulting in deviation from its normal eruption path and eventual impaction [8]. In contrast, the genetic theory attributes maxillary canine impaction to hereditary factors, supported by its familial aggregation and frequent association with other genetically determined dental anomalies, such as tooth agenesis, microdontia, and delayed eruption [9, 10]. Maxillary canines play a crucial role in facial esthetics, dental arch development, and occlusal function [11]. Miller [12] reported a strong association between congenital anomalies of the maxillary lateral incisors and the occurrence of palatally impacted canines.

Early identification or prediction of maxillary canine impaction is critical to prevent irreversible complications, particularly external root resorption of adjacent teeth. Timely orthodontic intervention may reduce treatment duration, complexity, and cost, underscoring the importance of early diagnosis. Jacobs [13] distinguished between early diagnosis, established before 10 years of age, and diagnosis made at a later stage. Before age 10, diagnosis is primarily based on family history and clinical findings, including the absence, reduced size, or peg‐shaped morphology of the maxillary lateral incisors [14]. After age 10, additional clinical and radiographic signs may be evaluated, such as asymmetry on alveolar palpation, differences in eruption timing between hemiarches, abnormal inclination of the lateral incisors, and radiographic overlap of the canine crown over the lateral incisor root on panoramic radiographs [15].

When early signs of ectopic eruption are identified, interceptive treatment may be considered to prevent impaction and its sequelae. Selective extraction of the deciduous canine at 8–9 years of age has been proposed as an effective interceptive approach in noncrowded Class I cases. Ericson and Kurol [16] reported that extraction of the primary canine before age 11 resulted in spontaneous correction of the eruption path of the permanent canine in 91% of cases when the canine crown was located distal to the midline of the lateral incisor. When the crown was positioned mesial to the lateral incisor midline, the success rate decreased to 64%. If interceptive treatment is unsuccessful or contraindicated, several alternative therapeutic options may be considered. The preferred treatment involves surgical exposure of the impacted canine, followed by orthodontic traction to guide the tooth into the dental arch [17, 18]. Other treatment options include autotransplantation of the impacted canine, extraction with substitution by mesial movement of the first premolar, extraction followed by posterior segmental osteotomy, or prosthetic replacement of the missing canine [19–21]. Retention of the deciduous canine generally carries an unfavorable long‐term prognosis, as progressive root resorption typically occurs regardless of residual root length or crown esthetics.

From a clinical perspective, improving the identification of morphologic features associated with maxillary canine impaction is highly relevant because delayed diagnosis may result in more complex orthodontic‐surgical treatment, greater risk of damage to adjacent teeth, and increased biological and economic burden for the patient. Since treatment planning in these cases requires a comprehensive evaluation of both dental and skeletal relationships, the assessment of mandibular arch morphology may provide additional diagnostic information beyond the maxillary findings alone. A better understanding of the mandibular arch form could therefore contribute to earlier recognition of patients at risk, more accurate treatment planning, and the development of individualized interceptive and corrective strategies.

It is important to analyze the arch form because there is a correlation between the vertical growth pattern and the mandibular arch form [22]. The presence of additional skeletal or dentoalveolar discrepancies increases the complexity of treatment in patients with canine impaction [23–26].

It is important to analyze arch form because several studies have reported that maxillary canine impaction is associated with dentoalveolar changes involving the dental arches. Compared with subjects with physiologic eruption, patients with impacted maxillary canines may present reduced transverse maxillary dimensions, differences in arch length and arch perimeter, and localized space deficiency in the canine‐premolar region [27, 28]. Moreover, buccal impaction has more commonly been associated with crowding and insufficient space, whereas palatal impaction has frequently been discussed in relation to a more complex developmental background and associated dental anomalies [29]. Collectively, these observations support the hypothesis that maxillary canine impaction may be part of a broader morphologic pattern rather than an isolated local eruption disturbance. Therefore, the evaluation of mandibular arch form may add useful information to the morphologic assessment of these patients.

Several studies have investigated craniofacial characteristics associated with maxillary canine impaction, with particular emphasis on maxillary arch morphology [30, 31]. In contrast, the contribution of the mandible and mandibular arch form has received limited attention. Given the central role of the mandible in craniofacial growth and occlusal development, further evaluation of mandibular morphology in patients with maxillary canine impaction is warranted. Therefore, the aim of the present study was to assess the mandibular arch form in patients with maxillary canine impaction by comparing subjects with buccal impaction, palatal impaction, and a control group with physiologic canine eruption.

## 2. Materials and Methods

This study was reported in accordance with the Strengthening the Reporting of Observational Studies in Epidemiology (STROBE) guidelines for observational studies [32].

All procedures described in this research protocol were conducted in accordance with the principles of the Declaration of Helsinki and were approved by the Ethics Committee of the University. Written informed consent was obtained from the parents of all patients prior to their inclusion in the study.

The sample consisted of three groups are as follows:

– Group B: patients with a maxillary buccally impacted canine (15 males and 17 females; mean age, 12.2 ± 0.7 years).
– Group P: patients with a maxillary palatally impacted canine (16 males and 16 females; mean age, 12.3 ± 0.6 years).
– Group C: control group with physiologic canine eruption (16 males and 16 females; mean age, 12.4 ± 0.3 years).

The impacted canines were distributed between sectors III and IV, according to the Lindauer et al. [33] classification. Group B showed an equal distribution between the two sectors (16 canines in sector III and 16 in sector IV), whereas Group P included 15 canines in sector III and 17 in sector IV.

The groups were retrospectively enrolled from patients treated at the Department of Orthodontics, Foggia, Italy, in chronological order from March 2024 to October 2025.

The inclusion criteria were unilateral maxillary buccal or palatal canine impaction, a normodivergent facial pattern (30.5° ≤ SNGoMe ≤ 35.5°), age between 10 and 12 years, and skeletal and dental Class I malocclusion. Patients with a history of previous orthodontic treatment, mono‐ or bilateral posterior crossbite, craniofacial malformations, or temporomandibular disorders were excluded.

Maxillary canine impaction was diagnosed on the basis of radiographic evidence of inclusion. In all included cases, the root apices of the impacted canines were not completely closed.

A priori sample size calculation was performed using 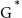
Power software (Version 3.1.9.2; Franz Faul, University of Kiel, Germany). Based on an assumed effect size of 0.40 [34], a multivariate analysis of variance (MANOVA) with three groups, a significance level (*α*) of 0.05, and a statistical power of 95%, the analysis indicated that a minimum sample size of 69 participants was required.

The choice of this effect size was based on previous methodological approaches reported in the literature on maxillary‐impacted canines and was considered appropriate [35, 36].

### 2.1. Cephalometric Analysis

Cephalometric analysis was performed on the lateral cephalograms. The analysis was carried out using the Dolphin Imaging software (Dolphin Imaging and Management Solutions, Chatsworth, CA, USA). Intraoperator reliability was assessed by repeating the cephalometric measurements after a 2‐week interval; and measurement error was calculated using Dahlberg formula.

The following cephalometric variables were analyzed: the divergence angle (SNGoMe) and the total gonial angle (ArGoMe). Cephalometric measurements were performed by an experienced orthodontist in a blinded manner.

### 2.2. Morphometric Measurements

The STL file used for morphometric measurements was obtained by intraoral digital scanning with a TRIOS intraoral scanner (3Shape, Copenhagen, Denmark) according to the manufacturer’s recommended protocol. For mandibular acquisition, the occlusal surfaces were scanned first, followed by the lingual and buccal surfaces. Areas showing incomplete data capture were subsequently rescanned to ensure the accurate visualization of all surfaces.

**Figure 1:**
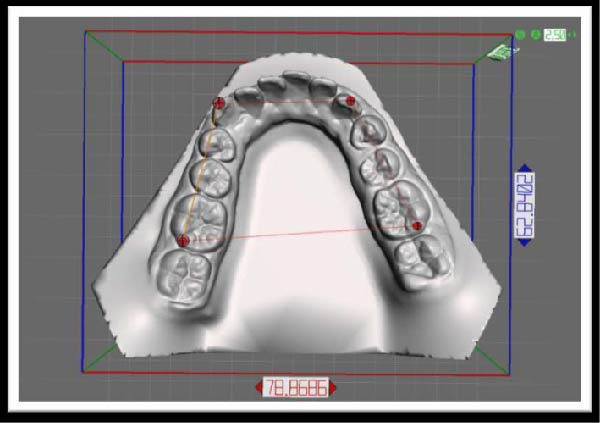
The reference polygon used in the morphometric analysis.

The STL file of the mandibular arch obtained from intraoral digital scanning was imported into Meshmixer software (Autodesk, San Rafael, Calif). The three‐dimensional model was reoriented to an occlusal view to standardize spatial positioning. Four anatomic landmarks were then identified to construct a reference polygon (Figure 1): the distobuccal cusp of the left permanent first molar, the distobuccal cusp of the right permanent first molar, the cusp tip of the right canine, and the cusp tip of the left canine.

The primary outcome variables were the anterior angle sum and posterior angle sum of the reference polygon. Specifically, the anterior angle sum was defined as the sum of the two anterior polygon angles corresponding to the canine landmarks, whereas the posterior angle sum was defined as the sum of the two posterior polygon angles corresponding to the first molar landmarks.

All measurements were performed twice by the same operator, with a 2‐week interval between the two assessments, to reduce the risk of measurement error. The operator was blinded to the group allocation to minimize the risk of measurement bias.

Random error was calculated using Dahlberg formula *(S* = √∑*d* ^2^/2*N*), where *d* represents the difference between the first and second measurements and *N* represents the number of measurements evaluated [37, 38]. The measurement error ranged from 0.02° to 0.03° for angular values.

### 2.3. Statistical Analysis

Data were analyzed using GraphPad Prism software 6.0 (GraphPad Prism Software, San Diego, CA, USA). Descriptive statistics were calculated for all variables of the groups (Table 1). Data distribution was assessed using the Shapiro–Wilk normality test. Homogeneity of variances was evaluated using Levene’s test, while the homogeneity of variance‐covariance matrices was assessed with Box’s *M* test. A MANOVA was conducted to investigate differences among the three groups (Table 2). Given the violation of the homogeneity of covariance matrices assumption (Box’s *M* test, *p* < 0.001), Pillai’s trace was selected as the primary multivariate test statistic due to its robustness to such violations. When a significant multivariate effect was detected, follow‐up univariate ANOVA was performed for each dependent variable (Table 3). Post hoc pairwise comparisons were conducted using Tukey’s honestly significant difference (HSD) test due to the homogeneity of variances (Table 4). Statistical significance was set at *p* < 0.05 for all analyses.

**Table 1:** Descriptive statistics of the sample.

Variables	Group	Mean (±SD)	Median	Maximum	Minimum	Passed normality test
ArGoMe	Group P Group B Group C	123.35 ± 7.67 128.59 ± 8.83 127.47 ± 6.77	122 128.65 126.25	133.8 151.5 145.5	108.1 112.8 112.4	Yes Yes Yes
SNGoMe	Group P Group B Group C	31.35 ± 1.38 31.34 ± 1.76 31.89 ± 1.34	32.3 31.36 32.71	33.1 32.1 33.23	30.7 29.58 30.55	No Yes Yes
Anterior angles sum	Group P Group B Group C	133.41 ± 6.97 123.68 ± 11.17 135.07 ± 6.65	134.65 124.1 135.29	145.52 144.58 148.22	115.42 100.98 122.99	No Yes Yes
Posterior angles sum	Group P Group B Group C	217.47 ± 8.24 226.07 ± 7.32 225.25 ± 6.95	218.37 224.81 224.34	233.84 241.93 237.79	202.12 215.26 211.82	Yes Yes Yes

**Table 2:** Multivariate analysis of variance (MANOVA).

Effect	Statistic	Value	*F*	*df*	*p* value
Groups	Pillai’s trace	0.520	7.997	8, 182	<0.001

*Note:* Pillai’s trace was used due to violation of the homogeneity of covariance matrices assumption (Box’s *M* test, *p* < 0.001).

**Table 3:** Univariate ANOVA.

Variable	*F*	*df*	*p* value	Partial *η* ^2^
ArGoMe	4.586	2.93	0.013	0.090
SNGoMe	0.564	2.93	0.571	0.012
Anterior angle sum	16.675	2.93	<0.001	0.264
Posterior angle sum	13.363	2.93	<0.001	0.223

**Table 4:** Tukey’s HSD post hoc test.

Dependent variable	Group (I)	Group (J)	Mean difference (I–J)	Std error	Sig.	95% confidence interval	
						Lower bound	Upper bound
ArGoMe	C	P	4.46	1.81	0.04	0.119	8.81
	C	B	1.11	1.95	0.835	−3.531	5.769
	P	B	−5.58	1.95	0.014	0.937	10.23
SNGoMe	C	P	0.41	1.64	0.965	−3.54	4.38
	C	B	−1.06	1.18	0.644	−3.93	1.79
	P	B	−1.48	1.44	0.560	−4.99	2.02
Anterior angle sum	C	P	1.67	1.7	0.591	−2.41	5.76
	C	B	11.39	2.29	0.000	5.83	16.94
	P	B	9.71	2.13	0.000	−14.79	−4.64
Posterior angle sum	C	P	7.77	1.83	0.000	3.4	12.15
	C	B	−0.81	1.83	0.896	−5.19	3.46
	P	B	−8.59	1.83	0.000	−12.97	−4.22

Abbreviations: B, buccal impaction; C, control group; P, palatal impaction.

## 3. Results

Table 1 summarizes the descriptive statistics for all variables analyzed in the three study groups. ARGoMe was 123.35 ± 7.67 in Group P, 128.59 ± 8.83 in Group B, and 127.47 ± 6.77 in Group C. SNGoMe was 31.35 ± 1.38 in Group P, 31.34 ± 1.76 in Group B, and 31.89 ± 1.34 in Group C. The sum of the anterior angles was 133.41 ± 6.97 in Group P, 123.68 ± 11.17 in Group B, and 135.07 ± 6.65 in Group C. The sum of the posterior angles was 217.47 ± 8.24 in Group P, 226.07 ± 7.32 in Group B, and 225.25 ± 6.95 in Group C.

MANOVA revealed a statistically significant overall effect of group membership on the combined dependent variables (ArGoMe, SNGoMe, anterior angle sum, and posterior angle sum). Based on Pillai’s trace, a significant multivariate effect was observed (*V* = 0.520; *F* = 7.997; *df* = 8, 182; *p* < 0.001), indicating that the three groups differed significantly when the variables were considered jointly.

Subsequent ANOVA tests demonstrated statistically significant differences among groups for ArGoMe (*F* = 4.586, *p* = 0.013), anterior angle sum (*F* = 16.675, *p* < 0.001), and posterior angle sum (*F* = 13.363, *p* < 0.001). No statistically significant differences were found among groups for SNGoMe (*F* = 0.564, *p* = 0.571).

Post hoc analyses demonstrated that, for the ArGoMe angle, the palatal impaction group exhibited significantly lower values than the buccal impaction group (−5.58; *p* = 0.014), whereas no significant differences were observed between the control group and either impaction group. For the anterior angle sum, the buccal impaction group showed significantly lower values than both the control group and the palatal impaction group (−11.39 and −9.71, respectively; *p*  < 0.001 for both comparisons). Regarding the posterior angle sum, the palatal impaction group presented significantly lower values than both the control group (7.77; *p*  < 0.001) and the buccal impaction group (8.59; *p*  < 0.001).

## 4. Discussion

### 4.1. Main Findings

The present study showed significant differences in mandibular arch morphology between patients with impacted maxillary canines and subjects with physiologic eruption after accounting for sagittal skeletal relationships and the vertical facial pattern. Using an angular morphometric analysis of digitally acquired models, these findings indicate that variations in mandibular arch shape are associated with maxillary canine impaction and suggest that the assessment of interarch morphology may complement conventional skeletal and maxillary evaluations in the characterization of patients with impacted canines.

The significant multivariate effect observed in the present study indicates that mandibular angular variables discriminate among vestibular impaction, palatal impaction, and control groups. Notably, these differences were not accompanied by significant variation in SNGoMe, whose mean values remained comparable across groups (31.35 ± 1.38 in Group P, 31.34 ± 1.76 in Group B, and 31.89 ± 1.34 in Group C). This suggests that the mandibular arch‐shape differences identified here are unlikely to reflect global vertical skeletal variation and may instead be related to more localized dentoalveolar or developmental factors. This interpretation is consistent with previous evidence showing measurable craniofacial differences in impacted‐canine patients despite the absence of marked skeletal disharmony. In particular, Ciavarella et al. [39] reported greater mandibular length in impacted‐canine patients than in controls (GO‐Me: 76.71 ± 5.20 mm vs. 73.80 ± 6.11 mm), together with higher SNA values (80.86° ± 3.29° vs. 80.22° ± 3.30°) and lower interclinoid distance (7.75 ± 1.78 mm vs. 9.36 ± 1.63 mm). Likewise, Tepedino et al. [40] found that patients with palatally displaced canines had a significantly smaller interclinoid distance (3.2 ± 1.2 mm vs. 5.3 ± 2.2 mm) and a greater SNA angle (86.0° ± 4.7° vs. 82.8° ± 4.8°) than matched controls. Together, these data support the concept that canine impaction is associated with a broader morphologic phenotype rather than with an isolated eruption disturbance.

### 4.2. Differences According to the Site of the Impaction

Differences in mandibular morphologic patterns were observed according to the site of impaction. Patients with palatal impaction exhibited significantly lower ArGoMe values (123.35 ± 7.67) and reduced posterior angle sums (217.47 ± 8.24) compared with patients with buccal impaction (128.59 ± 8.83 and 226.07 ± 7.32, respectively) and control subjects (127.47 ± 6.77 and 225.25 ± 6.95, respectively). These findings indicate a distinct mandibular arch configuration in cases of palatal impaction. Palatally impacted canines have been reported to be associated with more complex etiologic backgrounds, including genetic factors, altered eruption guidance, and associations with other dental anomalies [28, 41].

Previous quantitative evidence also supports the presence of a distinct craniofacial pattern in subjects with palatally impacted canines. Athanasiou et al. [41] reported a less convex facial profile, a more brachyfacial skeletal pattern, and a sagittally extended premaxilla, with effect sizes ranging from *η* ^2^ = 0.136 to 0.397 in females and from *η* ^2^ = 0.125 to 0.396 in males. More specifically, palatal canine impaction explained 39.7% of maxillary shape variation and 17.4% of mandibular shape variation in females and 39.6% and 31.6%, respectively, in males. Quantitative support for a distinct morphologic pattern in palatal canine impaction was also provided by Al‐Nimri and Gharaibeh [42], who found greater maxillary transverse dimensions in affected subjects than in controls, with an interpremolar width of 35.30 mm versus 33.00 mm and an intermolar width of 45.29 mm versus 43.65 mm, respectively, without significant differences in arch length–tooth size discrepancy or total tooth width. Although these studies focused mainly on maxillary and craniofacial morphology rather than mandibular angular configuration, their quantitative findings are in agreement with the present results in supporting the concept that palatal canine impaction is associated with a distinct morphologic pattern rather than with a random variation of the arch form.

In contrast, patients with buccally impacted canines exhibited significantly lower anterior angle sums (123.68 ± 11.17) compared with both the palatal impaction group (133.41 ± 6.97) and control subjects (135.07 ± 6.65). Buccal canine impaction has been quantitatively associated with localized space deficiency and reduced anterior transverse dimensions. Yan et al. [43] reported significantly smaller interpremolar width and maxillary skeletal width in subjects with buccally impacted canines than in both controls and subjects with palatal impaction, with interpremolar widths of 35.70 ± 2.06, 37.29 ± 2.29, and 36.87 ± 2.43 mm, respectively, and J‐J widths of 73.86 ± 4.37 mm, 76.99 ± 2.87 mm, and 77.42 ± 5.09 mm, respectively. In the same study, buccal impaction was mainly associated with anterior transverse dental and skeletal deficiency, whereas palatal impaction was mostly associated with small or missing lateral incisors. Although those measurements refer to transverse maxillary dimensions rather than mandibular angular morphology, they are consistent with the present findings in suggesting that buccal canine impaction is associated with a specific morphologic pattern related to spatial deficiency.

The absence of significant intergroup differences in the divergence angle in the present study suggests that, within the normodivergent sample examined, canine impaction was not associated with variations in the vertical facial pattern. This finding should, however, be interpreted in light of previous studies reporting different distributions of sagittal and vertical skeletal patterns in impacted‐canine patients. For example, Al Balbeesi et al. [44] found a higher frequency of canine impaction in subjects with Class III skeletal discrepancy, corresponding to 44.4% according to the Wits appraisal and 33.4% according to the ANB angle. The same study also reported an overall prevalence of hyperdivergence of 51.1%, with hyperdivergent patterns being more frequent in females and hypodivergent patterns being more frequent in males. These differences may reflect variations in sample composition, ethnicity, and study design.

### 4.3. Etiological Considerations

From an etiologic perspective, the present findings are consistent with previously proposed multifactorial models that incorporate genetic susceptibility, developmental disturbances, and spatial factors. Impacted canines have been reported to frequently coexist with other dental anomalies, including agenesis or microdontia of the maxillary lateral incisors, delayed eruption, and tooth transposition, suggesting a shared developmental background [45]. Within this context, the mandibular arch‐shape differences observed in the present study may be considered part of the associated morphologic characteristics rather than a secondary consequence of maxillary canine impaction.

### 4.4. Clinical Implications

The clinical relevance of the present findings lies in the observation that distinct mandibular morphologic patterns were associated with buccal and palatal canine impaction. This supports the inclusion of mandibular arch assessment in the diagnostic work‐up of patients with impacted maxillary canines, together with established clinical and radiographic findings, in order to improve the evaluation of interarch relationships and support more individualized treatment planning. In particular, palatal impaction was associated with significantly lower ArGoMe values and reduced posterior angle sums, whereas buccal impaction was characterized by significantly lower anterior angle sums compared with both the palatal impaction and control groups. These intergroup differences suggest that the location of maxillary canine impaction may be associated with distinct mandibular arch configurations. Maxillary canine impaction is a frequent and complex clinical condition, often associated with external root resorption of adjacent teeth, periodontal complications, prolonged treatment duration, and greater biomechanical demands. Although interceptive extraction of the deciduous canine may promote spontaneous eruption in selected cases, treatment outcomes remain variable. In this context, mandibular arch morphology may provide an additional morphologic parameter for the overall assessment of impacted‐canine patients.

### 4.5. Methodological Considerations

The digital workflow used in the present study supports the feasibility of the proposed analytic approach. Intraoral scanning combined with STL‐based angular morphometric analysis has been reported as an accurate and reproducible method for assessing dental arch morphology, with reliability comparable to that of conventional plaster models. In addition, the noninvasive nature of this approach is suitable for use in pediatric patients and allows its incorporation into orthodontic diagnostic protocols.

### 4.6. Limitations and Future Perspectives

Given the retrospective design of the study, the influence of unmeasured variables on the observed findings cannot be excluded, and no causal relationship or temporal sequence can be established. In addition, the restricted age range of the sample limits the generalizability of the findings to other age groups. Similarly, since only Class I normodivergent subjects were included, the present results may not be directly applicable to patients with different sagittal or vertical skeletal patterns, such as Class II, Class III, hyperdivergent, or hypodivergent subjects. Another limitation is that interexaminer reliability was not assessed. However, to reduce measurement error, all cephalometric and morphometric measurements were repeated twice in a blinded manner, and random error was calculated using the Dahlberg formula. Finally, although three‐dimensional data acquisition was achieved through digital intraoral scanning, the morphometric evaluation was based on a two‐dimensional angular analysis of the digital models. A fully three‐dimensional analytical approach may provide additional information on craniofacial morphologic characteristics and interarch relationships.

Further research is warranted to determine the extent to which mandibular arch morphology may serve as an adjunctive marker in the early diagnosis of maxillary canine impaction. In particular, more studies are needed to establish whether the morphologic differences observed in the present sample precede the impaction process and may therefore have predictive value during the mixed dentition. Future investigations should also explore the integration of digital mandibular morphometric analysis with three‐dimensional craniofacial assessment and conventional clinical‐radiographic parameters in order to refine diagnostic protocols and improve risk stratification. Moreover, the inclusion of subjects with different skeletal and growth patterns may help to clarify whether the observed associations are specific to normodivergent patients or reflect more general morphologic traits of impacted‐canine subjects. Such advances may ultimately contribute to more individualized treatment planning and to the optimization of interceptive and orthodontic management.

## 5. Conclusion

Mandibular arch morphology differed between patients with impacted maxillary canines and subjects with physiologic canine eruption, even when sagittal skeletal relationships and the vertical facial pattern were controlled. Distinct mandibular morphologic patterns were observed according to the site of impaction, with palatal and buccal impactions showing different angular configurations. These findings indicate that mandibular arch morphology is associated with maxillary canine impaction and suggest that mandibular arch assessment may provide complementary morphologic information when evaluating patients with impacted canines.

## Author Contributions

Conceptualization: Mauro Lorusso and Luigi Pio Marra. Methodology, data curation: Michele Tepedino. Software, writing – original draft preparation: Mauro Lorusso. Validation: Lucio Lo Russo. Formal analysis: Fariba Esperouz. Investigation, resources: Elena D’Angelo. Writing – review and editing: Domenico Ciavarella and Lucio Lo Russo. Visualization: Luigi Pio Marra. Supervision: Domenico Ciavarella. Project administration: Lucio Lo Russo.

## Funding

This research received no external funding. Open access publishing facilitated by Universita degli Studi di Foggia, as part of the Wiley ‐ CRUI‐CARE agreement.

## Ethics Statement

All the procedures of this research protocol adhered to the Declaration of Helsinki and were approved by the Ethics Committee of the University of Foggia (Approval Number 17/CE/2024). Informed consent was obtained from all individual participants included in the study.

## Conflicts of Interest

The authors declare no conflicts of interest.

## Supporting Information

Additional supporting information can be found online in the Supporting Information section.

## Supporting information


**Supporting Information 1** RAW DATA: Excel file containing the study’s raw data.

**Supporting Information 2** STROBE checklist: STROBE guidelines used for the study.

## Data Availability

The data that support the findings of this study are available from the corresponding author upon reasonable request.
